# Nuclear corepressor SMRT is a strong regulator of body weight independently of its ability to regulate thyroid hormone action

**DOI:** 10.1371/journal.pone.0220717

**Published:** 2019-08-12

**Authors:** Hiroaki Shimizu, Yu Lu, Kristen R. Vella, Federico Damilano, Inna Astapova, Izuki Amano, Megan Ritter, Molly R. Gallop, Anthony N. Rosenzweig, Ronald N. Cohen, Anthony N. Hollenberg

**Affiliations:** 1 Division of Endocrinology, Diabetes and Metabolism, Beth Israel Deaconess Medical Center and Harvard Medical School, Boston, Massachusetts, United States of America; 2 Division of Endocrinology, Diabetes and Metabolism, Weill Cornell Medicine, New York, New York, United States of America; 3 Division of Cardiology Massachusetts General Hospital, Boston, Massachusetts, United States of America; 4 Section of Endocrinology, Diabetes and Metabolism, University of Chicago, Chicago, Illinois, United States of America; Universidade do Estado do Rio de Janeiro, BRAZIL

## Abstract

Silencing Mediator of Retinoid and Thyroid Hormone Receptors (SMRT) and the nuclear receptor co-repressor1 (NCoR1) are paralogs and regulate nuclear receptor (NR) function through the recruitment of a multiprotein complex that includes histone deacetylase activity. Previous genetic strategies which deleted SMRT in a specific tissue or which altered the interaction between SMRT and NRs have suggested that it may regulate adiposity and insulin sensitivity. However, the full role of SMRT in adult mice has been difficult to establish because its complete deletion during embryogenesis is lethal. To elucidate the specific roles of SMRT in mouse target tissues especially in the context of thyroid hormone (TH) signaling, we used a tamoxifen-inducible post-natal disruption strategy. We found that global SMRT deletion causes dramatic obesity even though mice were fed a standard chow diet and exhibited normal food intake. This weight gain was associated with a decrease in energy expenditure. Interestingly, the deletion of SMRT had no effect on TH action in any tissue but did regulate retinoic acid receptor (RAR) function in the liver. We also demonstrate that the deletion of SMRT leads to profound hepatic steatosis in the setting of obesity. This is unlike NCoR1 deletion, which results in hepatic steatosis due to the upregulation of lipogenic gene expression. Taken together, our data demonstrate that SMRT plays a unique and CoR specific role in the regulation of body weight and has no role in TH action. This raises the possibility that additional role of CoRs besides NCoR1 and SMRT may exist to regulate TH action.

## Introduction

Nuclear receptor corepressors (CoRs) play important roles in the regulation of target gene transcription. While the function of CoRs was thought to be primarily subservient to the multiple nuclear receptors they interacted with recent studies have shown that CoRs themselves play larger roles in the specific regulation of DNA transcription and act as gatekeepers of homeostasis[1–3]. Classically, in the absence of ligand or the presence of a nuclear receptor (NR) -antagonist, CoRs bind to the NR and recruit a multi-protein complex that includes histone deacetylase3 (HDAC3), Upon the formation of CoR/NR/HDAC3 complex, HDAC3 is activated and represses gene transcription. However, many recent studies demonstrate that CoRs function in the both the presence and absence of agonist ligands and that the amount of CoR present actually determines the ligand-response. Indeed, the response to TH is dictated by the relative amount of functional CoR availability such that tissues become more responsive to TH in the absence of available CoR[4–6].

The two principal NR CoRs include Silencing Mediator for Retinoid and Thyroid Hormone Receptors (SMRT or NCoR2) and nuclear receptor corepressor1 (NCoR1). These paralogs are highly homologous though derived from separate genes[2]. Because they function as key transcriptional regulators, both have been genetically targeted in a variety of a models that have broadened the current understanding that both NCoR1 and SMRT function to regulate metabolic homeostasis[1, 7–9]. However, they clearly have distinct biological functions to regulate metabolism in target tissues[10, 11]. Indeed, our recent studies have demonstrated that NCoR1 plays a specific role in mediating TH action both in the liver and systemically whereas SMRT plays little to no role in TH action. However, a full analysis of SMRT’s role in TH action has been limited to the liver and the hypothalamic-pituitary-thyroid (HPT) axis[11].

Visceral obesity, including hepatic steatosis, is defined by abnormal fat accumulation that can lead to insulin resistance, non-alcoholic fatty liver disease (NAFLD) and metabolic syndrome. Importantly, hepatic deletion of both NCoR1 function and SMRT leads to substantial hepatic steatosis demonstrating that both CoRs cooperatively act to suppress hepatic lipid synthesis. SMRT has a distinct role in suppressing specific retinoic acid receptor (RAR) target genes involved in the regulation of lipid metabolism[11]. A number of studies show possible anti-obesity effects of natural or artificial retinoid derivatives through the suppression of lipid accumulation in multiple tissues, which suggests an anti-obesity role for SMRT[12–15]. However, due to the embryonic lethality of SMRT, the mechanism by which global SMRT deletion affects both lipid metabolism and retinoid signaling *in vivo* has yet to be fully understood.

To investigate the physiologic roles of SMRT in global TH action and metabolism, we expanded on a tamoxifen-inducible whole-body gene deletion strategy that we used previously[11]. Here, we show that global deletion of SMRT in adult mice leads to the development of visceral obesity that is independent of a high fat diet or increased food intake. This phenotype is associated with the alteration of RAR signaling in target organs such as the liver but has no effect on TH-signaling. Interestingly, global SMRT deletion causes hepatic steatosis via the activation of the obesity seen rather than through the *de novo* activation of lipogenic gene transcription as is seen in the hepatic specific deletion of NCoR1. These data indicate that SMRT plays little role in global TH-signaling but appears to regulate a significant to-be-defined pathway that is critical to body weight regulation.

## Materials and methods

### Mice

To generate the mouse strain with a post-natal global deletion of SMRT (SMRT^loxP/loxP^ UBC ^ERT2-Cre^; UBC-SKO mice), we used the same strategy as previously described[11, 16]. All experimental mice were on a C57BL6/129Svj mixed background. Livers from UBC-NCoR1ΔID mice were isolated as described[16].

### Animal experiments

All experiments were approved by the Beth Israel Deaconess Medical Center (BIDMC) Institutional Animal Care and Use Committee. Mice were single-cage housed in the BIDMC animal facility with a 12h light/dark cycle and supplied with standard chow diet except as indicated and water *ad libitum*. The experiments were performed in age- and sex-matched mice at 9 weeks of age unless otherwise specified. At this age, the UBC-SKO and SMRT^loxP/loxP^ (control) animals were treated with tamoxifen (20mg/100g BW) for 5 days as described previously [11, 16], and remained on standard chow diet (F6 rodent diet 8664; Harlan Teklad). At the end of the studies (at 21 weeks of age for the 1^st^ cohort of male mice, and at 24 weeks of age for both the 2^nd^ cohort of male mice and the wild type male mice for cardiac ultrasound study, at 4 weeks for the 3^rd^ cohort of male mice, and at 6 weeks for the female cohort of mice), all mice were sacrificed. Blood samples were taken by cardiac puncture. Tissues were rapidly collected, flash-frozen in liquid nitrogen, and stored at -80°C. The level of recombination and extent of SMRT deletion was assessed by quantitative PCR (qPCR) after the mice were euthanized. Only UBC-SKO mice with expression levels of the SMRT gene less than 75% of that were found in control animals were included in the experimental analysis.

### Western blotting

Protein lysates from target tissues and the samples for Western blot analysis were prepared as described previously[8, 11, 17]. Briefly, blots of 50 μg protein lysate were probed for SMRT-specific antibody (rabbit polyclonal anti-SMRTe antibody, 06–891; Millipore) or NCoR1-specific (rabbit polyclonal anti-NCoR antibody, A301145A; Bethyl), and then appropriate horseradish peroxidase (HRP)-conjugated secondary antibodies and visualized using ECL prime (GE Healthcare).

### Real-time quantitative PCR (RT-qPCR)

For mRNA expression analysis, RNA extraction, cDNA synthesis and qPCR were performed as described previously[11]. All mRNAs except *Chrebpα*, *Chrebpβ*, *Srebp-1c* were quantified using TaqMan Gene Expression Assays (Applied Biosystems) and TaqMan Universal PCR master mix, No Amp Erase (Applied Biosystems), in a total volume 10 μl. *Chrebpα*, *Chrebpβ*, *Srebp-1c*　mRNA expression levels were quantified using Power SYBR Green PCR master mix (Applied Biosystems) and previously published primers[18]. Relative mRNA levels of all genes were calculated using the standard-curve method and normalized to the level of cyclophilin A mRNA.

Cyclophilin A gene expression was assessed in all tissues of control and UBC-SKO mice (S2 Fig).

### Liver triglycerides and cholesterol

Lipids were extracted by the method described by Folch et al[8, 11, 19]. Extracted triglyceride and cholesterol contents were normalized by wet liver weight.

### Plasma lipids and hormone levels

Total serum cholesterol and triglycerides were measured using standard assays purchased from Stanbio Laboratory. Total plasma T4 (TT4) levels were measured using AccuDiag^™^ T4 ELISA Kit Cat# 3149–18 (Diagnostic Automation /Cortez Diagnostics, Inc. Calabasas, CA) in 25μl of serum, respectively. Circulating thyroid-stimulating hormone (TSH) was also measured in plasma via Milliplex MAP Mouse Pituitary Magnetic Bead Panel Mouse TSH only; EMD Millipore, Billerica, MA.

### Metabolic phenotyping

Whole-body energy metabolism was evaluated using a Comprehensive Lab Animal Monitoring System (CLAMS, Columbia Instruments). For 72 h measurements, mice were acclimated to the metabolic chambers for at least 48 hours to minimize stress from the housing change. CO_2_ and O_2_ levels were collected every 36 min for each mouse over the period of five experiment days. Energy Expenditure (EE), O_2_ consumption (VO_2_), CO_2_ production (VCO_2_), respiratory exchange ratio (RER), and food intake measurements were collected. During this period, mice were maintained at approximately 22–24°C under 12 hours light, 12 hours dark cycle. Food and water were available *ad libitum*.

### Core body temperature

Body temperature was measured weekly in the 2^nd^ cohort of mice by a digital rectal thermometer (Physi Temp Thermo Alert model TH-5; Physiotemp Instruments, Clifton, NJ).

### Food intake

A week prior to tamoxifen treatment, the 2^nd^ cohort of mice were single-cage housed and the amount of weekly food intake was measured.

### Body composition

4 weeks after tamoxifen treatment, mice were subjected to magnetic resonance imaging (MRI) using Echo MRI (Echo Medical Systems, Houston, TX) to determine body composition.

### Echocardiography studies

Echocardiography was performed on awake mice of the 1^st^ cohort using a Vivid 7 ultrasound system (GE Healthcare, IL) equipped with i13L probe (14 MHZ), after a three-day training. Parasternal short-axis images were recorded using both M-mode and 2D-mode imaging at the level of papillary muscles. Images were analyzed off-line using EchoPACS software (GE Healthcare). The average of at least three measurements was used for every data point from each mouse. Fractional shortening percentage was calculated as (LVIDd-LVIDs)/LVIDd*100. LVIDd: left ventricle internal diameter, diastole; LVIDs: left ventricle internal diameter, systole.

### Glucose tolerance test (GTT)

GTTs were performed on the mice 4 weeks and 12 weeks after tamoxifen treatment. Mice were fasted for approximately 14 hours in clean cages and their fasting blood glucose (FBG) was measured using a glucometer (OneTouch Ultra, Lifescan, Johnson & Johnson). Mice were then given an intraperitoneal injection of dextrose (1mg/g, Baxter Healthcare Corporation) and blood glucose was monitored over a 2 hour-time period.

### Insulin tolerance test (ITT)

At least 1week after the GTT, ITTs were performed on the same mice. Briefly, the mice were fasted for 5 hours in clean cages and fasting blood glucose (FBG) levels were measured. Insulin (0.5 units/kg, Humulin R, Eli Lilly) was then injected intraperitoneally and blood glucose was measured over a 2 hour-time period.

### Statistical analysis

Statistical analysis was performed using the Prism, Ver.6 program. All gene expression and hormonal analyses were performed on groups of 6–8 mice from each genotype. The differences in mRNA expression levels, serum glucose, hormone levels (insulin, leptin, TH and TSH), lipid profiles (triglycerides and total cholesterol concentration), and body composition were tested using unpaired Student’s t-test. Effects of tamoxifen treatment on different genotypes; such as BW, food intake, body core temperature, and results of cardiac ultrasound study were analyzed using Repeated Measures Two-way ANOVA with the Bonferoni *post hoc* test.

## Results

### Global SMRT deletion leads to obesity, glucose intolerance, and lower energy expenditure

**Fig 1:**
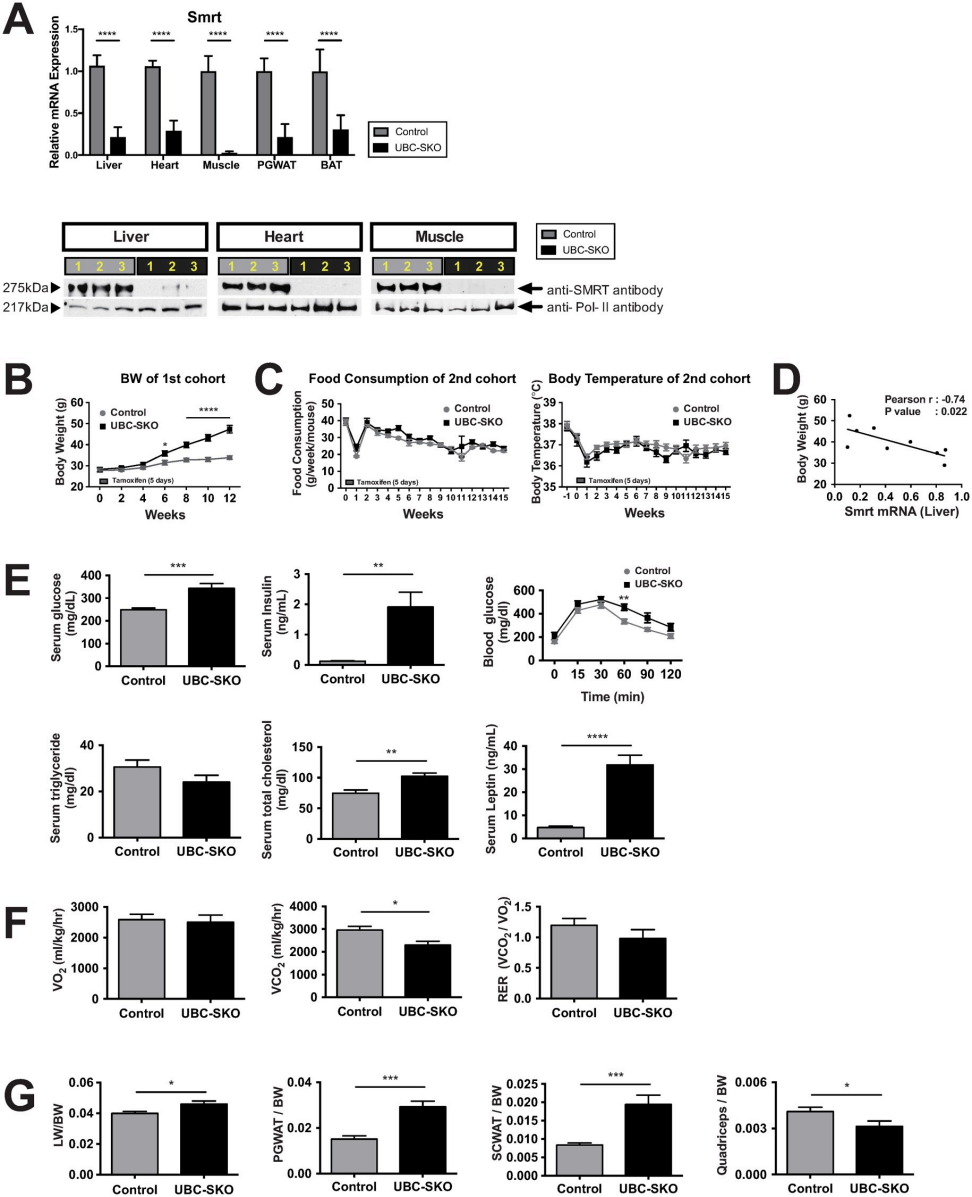
Global SMRT deletion leads to obesity, glucose intolerance, and lower energy expenditure. (A) We used qPCR analyses to determine the level of *Smrt* exon 11 expression in the liver, heart, muscle, PGWAT, and BAT at 12 weeks after tamoxifen treatment. Also Western blot analysis was used to determine the levels of SMRT protein using a SMRT specific antibody in the 1^st^ cohort of mice. Three samples were run in control mice (gray) and UBC-SKO mice (black). Blots were stripped and re-probed with anti-Pol-II as control. (B) Body weights in the 1^st^ cohort of mice starting at Day0 of tamoxifen administration through 12 weeks. (C) Food intake and body temperature were measured weekly (2^nd^ cohort). (D) We calculated the correlation between the levels of SMRT expression in the liver and body weight (2^nd^ cohort). (E) Serum glucose, insulin, glucose tolerance tests, serum triglyceride, total cholesterol, and leptin were measured at 12 weeks after tamoxifen treatment (2^nd^ cohort). (F) The 2^nd^ cohort mice were placed in a CLAMS apparatus and monitored for oxygen consumption (VO_2_), carbon dioxide consumption (VCO_2_), and Respiratory-expiratory ratio (RER) over 72 hours. All data were normalized to body weight. (G) The weights of liver, PGWAT, SCWAT, and quadriceps were normalized to body weight in the 2^nd^ cohort of mice. For qPCR data in panels A, and panels E-G (except the figure of the glucose tolerance test in panel E), the data were analyzed by unpaired t-test. In the analyses for BW (panel B), Food consumption (panel C), Body temperature (panel C), and glucose tolerance (panel E), Repeated Measures Two-way ANOVA was used. Results are shown as the mean±SEM, the p-value was shown as; ****, p< 0.0001; ***, p< 0.001; **, p< 0.01; *, p< 0.05. Panel D was analyzed by Pearson’s correlation coefficient analysis, and the p-value was shown as p = 0.022; *, p< 0.05. 1^st^ cohort included n = 6–7 mice/group, and 2^nd^ cohort included n = 6–8 mice/group.

To investigate the functional role of SMRT globally in all target organs and to avoid the embryonic lethality of the SMRT knockout mouse, we used a ubiquitously expressed tamoxifen-inducible Cre (UBC^ERT2-Cre^) to disrupt SMRT postnatally[11, 16]. As shown in the representative study design, all mice were administrated tamoxifen between 8–10 weeks of age (S1A Fig). Starting 1 week before tamoxifen treatment, we measured body weight, core body temperature and food intake weekly. 12 weeks after tamoxifen treatment, all mice were sacrificed. We confirmed that exon 11 of SMRT mRNA was deleted in a wide variety of tissues and that SMRT protein levels were minimally present in the heart, muscle and liver (Fig 1A). Starting 6 week after tamoxifen administration, UBC-SKO mice started to gain body weight on a standard chow diet with progressive development of obesity through the reminder of the experiment (Fig 1B). To exclude the possibility that the obesity of UBC-SKO mice (SMRT^loxP/loxP^ UBC^ERT2-Cre^) was caused by Cre-induced organ toxicity we set up a separate control mouse cohort comparing UBC^ERT2-Cre^ and Cre-negative littermates on a C57/BL6 background, which is more prone to obesity[20]. As shown S1B Fig, the administration of tamoxifen to UBC^ERT2-Cre^ and Cre-negative littermates did not increase body weight on a C57/BL6 background, indicating that the obese phenotype of UBC-SKO is specific to SMRT deletion (S1B Fig).

For further analysis of the obese phenotype, we set up a 2^nd^ cohort of male mice. Indeed, UBC-SKO mice in this cohort became obese starting 6 weeks after tamoxifen treatment as well (S1C Fig). Importantly the development of obesity was independent of changes in food intake or body temperature (Fig 1C). qPCR analysis of gene expression in the liver showed that the extent of SMRT exon11 truncation has a significant negative correlation with BW (Fig 1D). Furthermore, UBC-SKO mice were glucose intolerant and had significantly higher concentrations of serum glucose as well as insulin 12 weeks after tamoxifen treatment, consistent with insulin resistance (Fig 1E). Moreover, serum cholesterol was higher in UBC-SKO mice but serum triglyceride levels were comparable to control mice (Fig 1E). Additionally, UBC-SKO mice have a decrease of VCO_2_ and a slightly lower RER ratio compared with controls, indicating that they seem to have lower EE (Fig 1F). Consistent with this, UBC-SKO mice showed a profound obese phenotype with fatty liver and an increase in white adipose tissue (WAT); perigonadal WAT (PGWAT) and subcutaneous WAT (SCWAT). The decrease of quadriceps weight was also seen in UBC-SKO mice (Fig 1G).

To examine the mechanism of body weight regulation, we looked at serum leptin levels in UBC-SKO mice and resulting neuropeptide gene expression in the hypothalamus. As expected, the concentration of serum leptin in UBC-SKO mice was extremely high based on their fat mass (Fig 1E). However, mRNA levels of the gene encoding the orexigenic peptide; *Agrp*, and the gene encoding the anorexigenic peptide; *α-MSH (Pomc)* and its receptor; *Mc4r* were not different from controls consistent with central leptin resistance (S1D Fig).

Taken together, the global deletion of SMRT leads to significant obesity independent of a high fat diet or food intake.

### Global SMRT deletion does not affect thyroid hormone action

**Fig 2:**
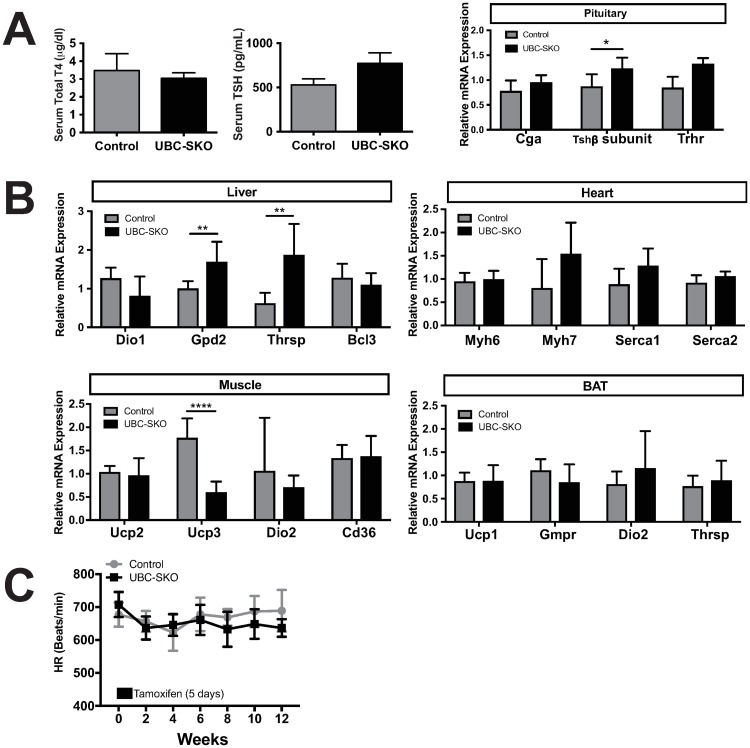
Global SMRT deletion does not affect the HPT axis or thyroid hormone action. (A) Serum total T4 and TSH were measured, and gene expression of *Cga*, *Tshβ* subunit, and *Trhr* in the pituitary were quantified by qPCR in control and UBC-SKO male mice 12 weeks after tamoxifen treatment (1^st^ and 2^nd^ cohort). (B) mRNA expression levels of T3-target genes in the liver, heart, skeletal muscle, and brown adipose tissue (BAT taken from 2^nd^ cohort mice) were quantified by qPCR. (C) Heart rate (HR) was measured weekly by cardiac echocardiography in the 1^st^ cohort mice weekly. For panels A and B, the data were analyzed by unpaired t-test. Panel C was analyzed by Repeated-Measures Two-way ANOVA. Results are shown as the mean±SEM, and the p-values as; ****, p< 0.001; **, p< 0.01; *, p< 0.05. 1^st^ cohort included n = 6–7 mice/group, and 2^nd^ cohort included n = 6–8 mice/group.

To determine the effect of SMRT deletion on TH action in target tissues, we first analyzed the HPT axis in control and UBC-SKO mice. Previously, we demonstrated that serum T4 levels in UBC-SKO mice were comparable to controls 4 weeks after tamoxifen administration[11]. Herein, we demonstrate that T4 levels remain unchanged between UBC-SKO and control mice 12 weeks after tamoxifen administration (Fig 2A). Although there is significant increase in *Tshβ* subunit mRNA expression in UBC-SKO mice, there was no difference in the serum TSH concentration (Fig 2A). Furthermore, pituitary TR target genes such as *Cga* and *Trhr* were not different between the two genotypes (Fig 2A). These data indicate that whole-body SMRT deletion has a minimal effect on the HPT axis.

Next, we performed analysis of TR target gene expression in different TH target tissues. While a few specific genes such as *Thrsp* and *Gpd2* were higher in the livers of UBC-SKO mice, a number of TR target genes (*Dio1* and *Bcl3*) were not significantly up-regulated in the absence of SMRT (Fig 2B). Additional analysis of different TR target organs including the heart, muscle, and brown adipose tissue (BAT) showed that there was little difference in gene expression between the two genotypes in context of TR target genes (Fig 2B). We also performed cardiac ultrasound on these groups and showed that there was no difference in the heart rate (HR) between UBC-SKO and control mice, indicating that SMRT deletion has no effect on TH sensitivity in the heart in the context of heart rate (Fig 2C). Further cardiac echo analysis showed that left ventricular internal dimension diastole (LVIDd) of UBC-SKO mice was comparable with the control group. However, there was a lower % fractional shortening (%FS) in UBC-SKO mice (S1E Fig). Interestingly, we do not believe this effect is SMRT specific as changes were seen in fractional shortening in mice independent of the amount of SMRT deleted. Taken together, these data show whole-body SMRT deletion neither plays a functional role in TR target organs nor changes the HPT axis.

### SMRT deletion actively enhances hepatic lipogenesis

**Fig 3:**
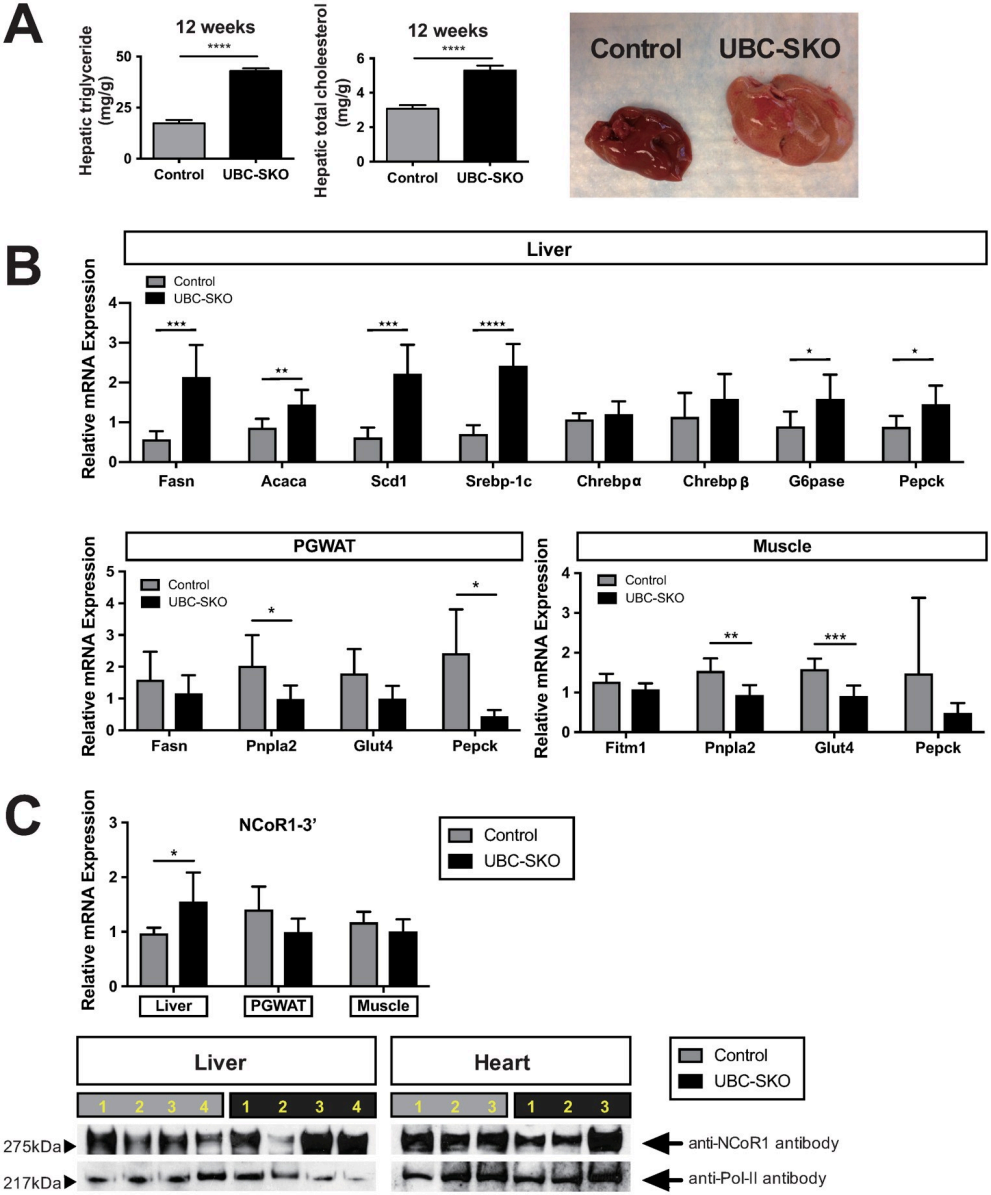
SMRT deletion actively enhances hepatic lipogenesis. (A) Hepatic triglyceride and cholesterol levels were measured in the 1^st^ cohort mice 12th weeks after tamoxifen treatment. Images of control and UBC-SKO mice the livers are shown. (B) The mRNA expression of lipogenic and gluconeogenic-related genes in the liver, PGWAT, and muscle (1^st^ cohort of mice) (C) mRNA expression of NCoR1 in the liver, skeletal muscle, and PGWAT were quantified by qPCR. Protein expression of NCoR1 in the liver and heart were assessed by Western blot, using a NCoR1 specific antibody (n = 3–4 animals per group). Control samples are indicated by gray lanes and UBC-SKO mice are indicated by black lanes. For panel A and all qPCR analyses in panels B, and C, the data were analyzed using an unpaired t-test. These results are shown as the mean±SEM, and the p-values as; ****, p< 0.001; ***, p< 0.001; **, p< 0.01; *, p< 0.05 (1^st^ cohort of mice, n = 6–7 mice/group).

For further analysis of organ function in the UBC-SKO mouse, we next focused on hepatic lipogenesis. Consistent with a heavier body weight UBC-SKO mouse had hepatomegaly, and hepatic lipid content was dominated by an accumulation of triglycerides (Fig 3A). Hepatic gene expression analysis showed that UBC-SKO mice had significant up-regulation of both lipogenic genes (*Fasn*, *Acaca*, and *Scd1*) and gluconeogenic-related genes (*G6pase*, and *Pepck*). Interestingly, the expression of *Srebp-1c*, which is a well-recognized transcription factor that regulates lipogenic genes, was significantly increased in the UBC-SKO mouse liver (Fig 3B). However, there was no change in the expression of the lipogenic transcription factors *Chrebpα and Chrebpβ*, which are regulated by the deletion of NCoR1 [11]. Analysis of lipogenic and gluconeogenic-related genes in PGWAT and muscle showed significant decreases in *Pnpla2* and *Pepck* in PGWAT and *Pnpla2* and *Glut4* in the muscle, suggesting that the function of SMRT is tissue specific (Fig 3B). Indeed, the mRNA level of NCoR1 in the UBC-SKO liver was up-regulated suggesting NCoR1 works in a compensatory manner. However, there was no change in the protein levels of NCoR1 in the tissues tested (Fig 3C). Moreover, the qPCR assessment of reference gene expression in each tissue demonstrated that minimally higher levels of *Cyclophilin-A* were seen in liver, heart, and muscle in UBC-SKO mice and similar levels in pituitary and PGWAT which do not affect interpretation of mRNA expression (S2 Fig). These data indicate that SMRT plays a significant role as a suppressor of liver lipogenesis in an adult mouse, and the presence of NCoR1 does not seem to compensate for the hepatic steatosis seen with SMRT deletion.

### Insulin resistance and fatty liver in UBC-SKO mouse are secondary to the progression of obesity

**Fig 4:**
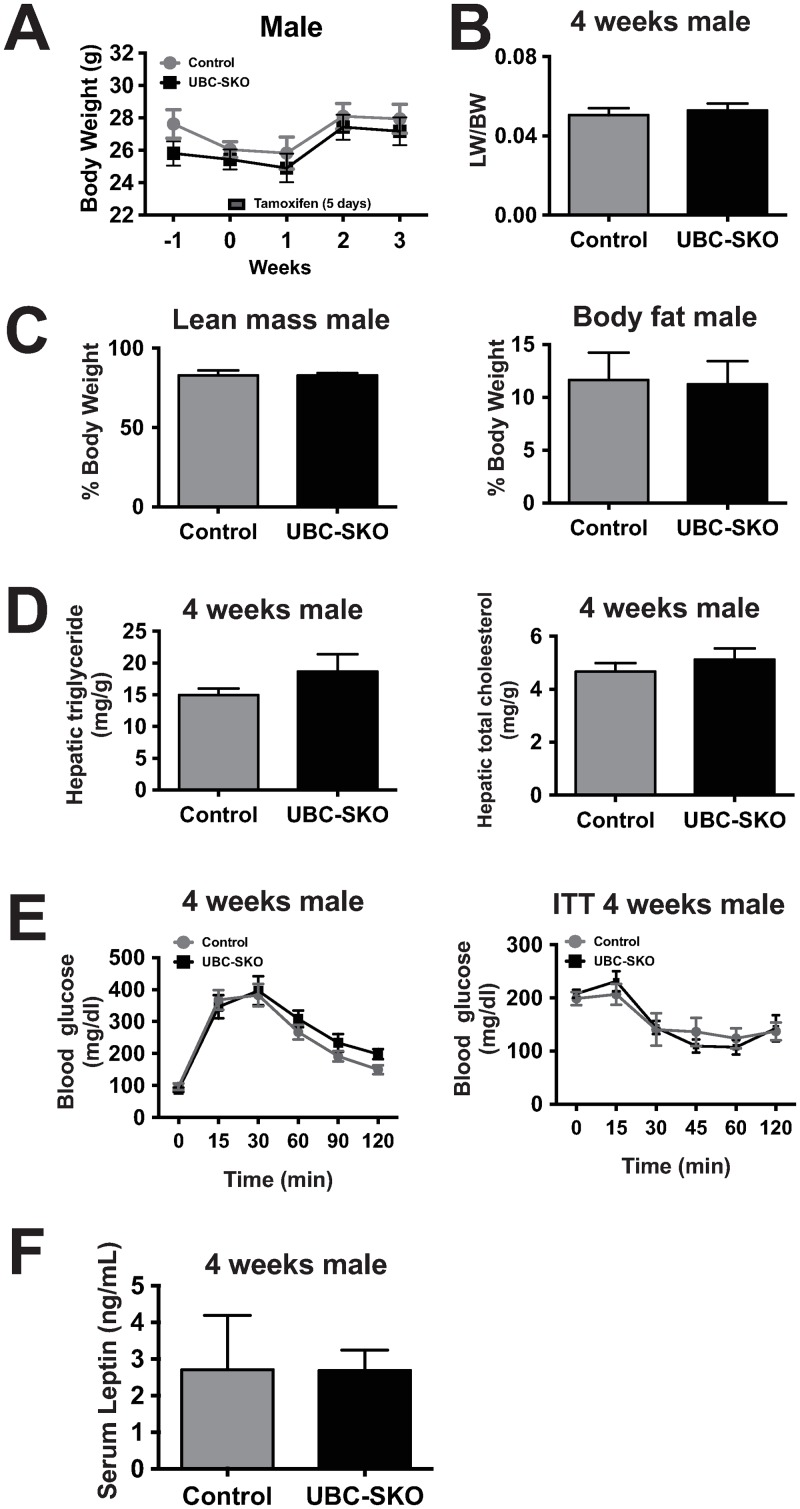
Insulin resistance and fatty liver in UBC-SKO mouse occur secondary to the progression of obesity. (A) Body weight of control and UBC-SKO mice were measured weekly for 3 weeks after tamoxifen treatment in the 3^rd^ male cohort. (B) Liver weights in control and UBC-SKO of male mice (3^rd^ cohort). (C) Body composition was measured via MRI in control and UBC-SKO male mice (3^rd^ cohort). (D) Hepatic triglyceride and cholesterol levels were measured in both control and UBC-SKO male mice (3^rd^ cohort). (E) Glucose tolerance and insulin tolerance tests were performed in male mice 4 weeks after tamoxifen treatment (3^rd^ cohort). (F) Serum leptin levels were measured in both control and UBC-SKO male mice (3^rd^ cohort). All results are shown as the mean±SEM. For panels A and E, Two-way Repeated-Measures ANOVA was used. For panels B, C, D, and F, the data were analyzed by unpaired t-test (3^rd^ male cohort n = 7–8 mice/group).

To better understand the mechanism that causes obesity in the UBC-SKO mouse, we generated a 3^rd^ cohort of (young) male mice and observed them through 4 weeks post-tamoxifen administration. As shown in Fig 4A and 4B, there was no increase in body weight or hepatomegaly in the UBC-SKO mice three weeks after tamoxifen treatment in the 3^rd^ cohort of mice (Fig 4A and 4B). Furthermore, MRI measurement of body composition showed that both lean mass and fat mass are similar between control mice and UBC-SKO mice (Fig 4C). Also, there were no differences detected in hepatic lipid composition, glucose tolerance, insulin tolerance, and leptin levels between UBC-SKO and control mice 4 weeks after tamoxifen administration (Fig 4D–4F). These data suggest that insulin resistance in male UBC-SKO mice is secondary to the progression of obesity.

To confirm that SMRT deletion has similar effects in females, we generated a female cohort of UBC-SKO mice. Beginning 4 weeks after tamoxifen treatment, female UBC-SKO mice have a significant increase in body weight over control mice (S3A Fig). Female UBC-SKO mice have normal liver weight, lean mass, body fat percentage, hepatic triglycerides, hepatic total cholesterol, glucose tolerance, insulin tolerance and leptin levels (S3B–S3F Fig). Taken together, these data further support the conclusion that female UBC-SKO mice are also prone to obesity. However, to prove whether the development of insulin resistance and fatty liver in female UBC-SKO mice might be caused by obesity, the further analysis of an older female cohort will be needed.

To determine the effect of SMRT deletion on lipogenic and gluconeogenic-related gene expression prior to the onset of obesity, we analyzed various tissues in the 3^rd^ male cohort (4 weeks post-tamoxifen) and female cohort (6 weeks post-tamoxifen). There were no differences in the expression of the vast majority of lipogenic and gluconeogenic genes in the livers of UBC-SKO and control mice in either cohort (S4A and S3B Figs). Interestingly, *Dio1* expression was decreased in both males and females and *Scd1* expression was slightly upregulated in females. While UBC-SKO mice had an increase in *Pepck* expression in SCWAT, other metabolic genes in SCWAT and PGWAT showed no difference between UBC-SKO and control mice in the 3^rd^ cohort of male (S4C and S3D Figs). We also examined EE in these mice in the early weeks after tamoxifen treatment, and confirmed that there were no differences between UBC-SKO and control mice in either cohort in the both light and dark illumination cycle (S4E Fig). Additionally, TH levels were similar between UBC-SKO and control mice in the 3^rd^ male cohort (S4F Fig). Thus, we can conclude that insulin resistance and alterations in lipogenic and gluconeogenic gene expression seen in the 1^st^ and 2^nd^ cohorts of male mice examined 12 weeks after tamoxifen injection occur secondary to obesity in the UBC-SKO mice.

### Global SMRT deletion predominantly activates RAR signaling in the liver

**Fig 5:**
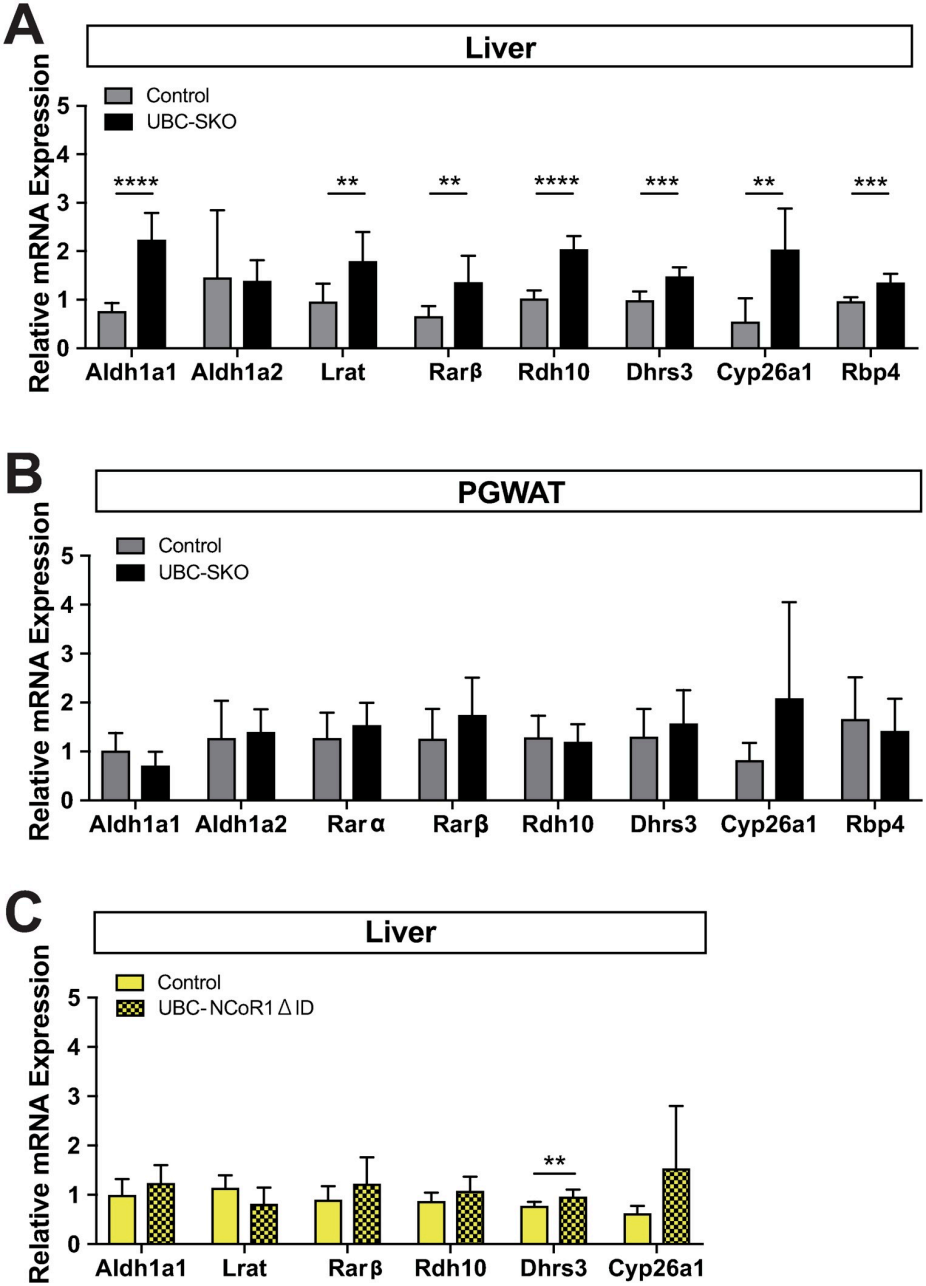
Global deletion of SMRT predominantly activates RAR signaling in the liver. (A) mRNA expression levels of the RAR signaling target genes in the liver of control and UBC-SKO mice 12 weeks after tamoxifen treatment were quantified by qPCR. (B) mRNA expression levels of RAR signaling target genes in PGWAT of control and UBC-SKO mouse were assessed by qPCR. (C) mRNA expression levels of RAR signaling target genes in the liver of control and global NCoR mutant (UBC-NCoR1ΔID) mice were assessed by qPCR. For panels A, B, and C, all qPCR data were analyzed by an unpaired t-test. These results are shown as the mean±SEM. ****, p< 0.0001; ***, p< 0.001; **, p< 0.01. The 1^st^ cohort mice (n = 6–7 mice/group) were used in panels A and B, and UBC-NCoR1ΔID mice (n = 6–8 mice/group) were used in panel C.

Previous *in vitro* studies have demonstrated that SMRT is preferentially recruited to RAR/RXR heterodimers[21, 22]. Similarly, *in vivo*, the deletion of SMRT in hepatocytes leads to the upregulation of specific RAR target genes (ex. *Cyp26a1*, *RARβ*)[11]. On the basis of these studies, we hypothesized that SMRT may play a dominant role in the regulation of RAR signaling globally. Thus we analyzed the expression of RA responsive genes in target tissues of the 1^st^ male cohort 12 weeks after tamoxifen treatment. As shown in Fig 5A, a variety of RAR target genes responsible for retinoid transport and catabolism showed significantly higher expression in the UBC-SKO mouse liver (Fig 5A). Interestingly, the expression levels of the same genes in PGWAT of UBC-SKO mice were comparable to controls (Fig 5B). These data suggest that SMRT regulation of RAR signaling is organ specific.

To determine if global NCoR1 disruption could also regulate RAR target genes we examined the livers from global NCoR1 mutant (UBC-NCoR1ΔID) mice[16]. This model expresses a hypomorphic NCoR1 allele that lacks the NR interacting domains. As expected, although there was higher *Dhrs3* gene expression seen, the majority of the additional RAR responsive genes studied were similar in expression to control (NCoR1^loxP/loxP^) mice (Fig 5C). Thus, the remaining NCoR1 in UBC-SKO mice likely cannot be recruited to RAR target genes in the liver.

## Discussion

Obesity has been recognized as world-wide public health concern and is highly linked to metabolic disorders including type 2 diabetes mellitus and dyslipidemia[23]. Mechanistically obesity is secondary to genetic and/or environmental changes via three processes; 1) food intake 2) energy expenditure and 3) adipogenesis[24, 25]. In each of these processes, a variety of signaling pathways have been identified as playing a role. Included in these pathways are those governed and regulated by the NR superfamily. Indeed, NRs are recognized as therapeutic targets for metabolic disorders and their agonists are clinically approved for therapeutic use[26].

Previous studies have demonstrated that NR function is enhanced by increasing the sensitivity of the receptors to their endogenous ligand through alterations in co-regulator expression[9, 27]. Thus, NR action is changed by the balance of co-regulators, and the amount of CoRs and coactivators (CoAs) present. By using strategies that change the interaction between the NR and co-regulator complex, with or without ligand, the metabolic functions of CoAs have been clarified[3, 28]. On the other hand, the global function of CoRs *in vivo* has not been extensively studied because of embryonic lethality[29–31].

Previous studies analyzing the role of SMRT *in vivo* have focused on: 1. tissue-specific approaches; 2. have utilized altered SMRT alleles that impair their interactions with NRs or 3. studied mice haploinsuffcient for the SMRT allele. Each of these models has disclosed a role for SMRT in metabolism[7, 11, 32]. In particular both the mice haploinsufficient for SMRT and mice with a targeted disruption of the first receptor interaction domain (RID) of SMRT have developed obesity, hepatic steatosis, and insulin resistance, but their phenotype only manifested in the context of a high fat diet (HFD)[7, 32, 33]. Importantly, previous models of SMRT deletion demonstrated little role for SMRT in mediating the actions of TH[11]. This is in contrast to its paralog NCoR1. Thus, to further study the specific function of SMRT, we used a tamoxifen-inducible post-natal disruption strategy as previously described[11, 16]. Prior to the data presented herein we had used the same model to determine whether SMRT was required to regulate the HPT axis in a similar fashion to NCoR1[16].

Importantly, this model allows us to ablate SMRT without affecting viability of the animals because it is done at 9–10 weeks of age.

Strikingly, we found that the UBC-SKO mouse develops significant obesity associated with both insulin resistance and glucose intolerance, and slightly decreased EE. Although there is the possibility that overproduction of leptin from expanded adipose tissue may alter food intake, this was not the case and may also indicate that the phenotype is related to a central nervous system process that likely controls EE. This will require further study but is likely critical given the extent of the obesity seen in this model on a normal chow diet. Importantly, the phenotype is seen in both male and female mice but appears to develop slowly.

In addition to the obesity seen, UBC-SKO mice also demonstrated profound hepatic steatosis. Previous work in our lab and others has demonstrated the importance of NCoR1 in suppressing lipogenesis[8, 10, 11, 17, 34]. Indeed, when NCoR1 or HDAC3 is specifically deleted in hepatocytes a lipogenic program is activated leading to steatosis without evidence of insulin resistance or glucose intolerance. In contrast, the specific deletion of SMRT from hepatocytes does not activate lipogenesis. Furthermore, global SMRT deletion does not affect lipogenic gene expression (4 weeks post-tamoxifen treatment). Rather, these genes are altered after the development of obesity, hepatomegaly, and glucose intolerance (12 weeks post-tamoxifen treatment). Indeed, while the deletion of NCoR1 activates lipogenesis through direct derepression of lipogenic targets and potentially by the activation of *Chrebp* the deletion of SMRT has little effect on these targets[11, 17, 18, 35]. In contrast, the global deletion of SMRT leads to the eventual activation *Srebp-1c*, a lipogenic transcription factor regulated in an insulin dependent manner, which is consistent with the development of insulin resistance[36, 37].

The other significant aim of these studies was to determine whether SMRT plays a role in the regulation of TH responsiveness in target tissues. Previous work from our laboratory has shown that NCoR1 preferentially regulates the *TRβ* isoform, which raises the possibility that SMRT interacts mainly with *TRα* in tissues such as the heart or skeletal muscle[6, 8, 16, 17, 38]. However, as demonstrated here, heart rate is not altered in mice that lack SMRT. Similarly, there was no evidence of activation of gene expression in other *TRα* signaling tissues such as skeletal muscle or brown adipose tissue. Thus, unlike NCoR1, SMRT appears to play little role in regulating the sensitivity of TH-responsive signaling pathways to TH. It remains to be determined whether an alternative CoR plays a role in these other tissues.

In contrast to its lack of action in regulating the response to TH, SMRT again showed a preference for RAR signaling. Indeed, we found higher expression of retinoid catabolic enzymes (ex. *Aldha1*, *Rdh10*, *Dhrs3*, and *Cyp26a1*) in the mouse liver of UBC-SKO mice, suggesting that retinoid catabolism seems to be enhanced by SMRT-deletion. This would be consistent with SMRT playing a role in RA-sensitivity at least in the liver. A recent report also showed that SMRT similarly activates RAR signaling in vertebral and axial development[31, 39]. It also remains possible that the increased sensitivity to RA seen in the liver could affect the obese phenotype seen. In previous studies, mice racking retinaldehyde dehydrogenase (*Raldh; Aldh*) were lean and had improved insulin sensitivity[40, 41]. These studies indicate the possibility that retinoid metabolites regulate adipogenesis and glucose metabolism. Measurement of tissue retinoid concentrations using LC-MS as well as the effects of RAR antagonists in SMRT-deficient mice will be needed to determine whether this hypothesis warrants further investigation.

In summary, the data presented herein demonstrate a unique and profound role for SMRT in body weight regulation. Determining the pathways involved will be critical as it remains possible that HDAC3 inhibition could lead to weight gain in the pharmacologic setting. Surprisingly, SMRT plays little role in TH action in any target cell type tested. These data raise the possibility that additional CoRs besides NCoR1 and SMRT may exist to regulate the *in vivo* actions of TH.

## Supporting information

S1 Fig The global deletion of SMRT *in vivo* causes obesity without any effect on cardiac function. (A) A schematic representation of study design: tamoxifen treatments were performed in all mouse cohorts between 9–10 weeks of age. CLAMS studies were performed 3-4weeks (3rd male and female cohort), 4–5 weeks (1^st^ and 2^nd^ cohort), and 9–10 weeks (1^st^ and 2^nd^ cohort) after tamoxifen treatment. (B) Body weights were measured weekly in both wild type (WT) and WT-Cre male mice for 12 weeks following tamoxifen treatment (n = 6–8 mice/ group). (C) Body weights were measured weekly in control and UBC-SKO mice (2^nd^ cohort). (D) Gene expression of *Smrt*, *NCoR1-3’*, *Pomc*, *Mc4r*, and *Agrp* in the hypothalamus were quantified by qPCR in control and UBC-SKO male mice at 12 weeks after tamoxifen treatment (2^nd^ cohort mice). (E) Analyses of LVIDd, % fractional shortening (%FS), and heart rate (HR) using cardiac echocardiography were performed on control and UBC-SKO mice (upper panels, 1^st^ cohort) and WT and WT-Cre (lower panels, n = 6–8 mice). For panels B, C and E, Two-way Repeated-Measures ANOVA was used, and for panel D, all qPCR data were analyzed by an unpaired t-test. Results are shown as the mean±SEM and p-values are; ****, p< 0.0001; ***, p< 0.001; **, p< 0.01; *, p< 0.05. 1^st^ cohort included n = 6–7 mice/group, and 2^nd^ cohort included n = 6–8 mice/group.
(PDF)

S2 Fig The mean Ct value of Cyclophilin-A mRNA expression per tissue type. The mean of CT value was analyzed by qPCR in each tissue. Data were analyzed by unpaired t-test. Results are shown as the mean±SEM, and the p-values as; ****, p< 0.0001; **, p< 0.01; *, p< 0.05 (1^st^ cohort, n = 6–7 mice/group).
(PDF)

S3 Fig Body weights increase in female UBC-SKO mice at 4 weeks of age. (A) Body weights were measured weekly in female control and UBC-SKO mice 6 weeks after tamoxifen treatment. (B) Liver weights, (C) body composition, and (D) hepatic triglycerides and cholesterol levels were measured in control and UBC-SKO mice. (E) Glucose tolerance and insulin tolerance tests were performed in female mice at 4 weeks after tamoxifen treatment. (F) Serum leptin levels were measured in control and UBC-SKO mice 6 weeks after tamoxifen treatment. All results are shown as the mean±SEM. For panels A and E, Two-way Repeated-Measures ANOVA was used, and the p-value is; **, p< 0.01; *, p< 0.05. For panels B, C, D, and F, the data were analyzed by unpaired t-test. The female cohort had n = 8–9 mice/group.
(PDF)

S4 Fig SMRT deletion does not affect gene expression, energy expenditure, and TH levels in the initial weeks after tamoxifen treatment. (A) In male mice, liver gene expression was measured in control and UBC-SKO mice 4 weeks after tamoxifen treatment (3rd cohort). (B) In female mice, liver gene expression in control and UBC-SKO female mice was assessed by qPCR 6 weeks after tamoxifen treatment (female cohort). (C) In male mice, gene expression in SCWAT was measured in control and UBC-SKO mice 4 weeks after tamoxifen treatment (3rd cohort). (D) In male mice, gene expression in PGWAT was measured in control and UBC-SKO mice 4 weeks after tamoxifen treatment (3rd cohort). (E) Carbon dioxide production (VCO_2_), oxygen consumption (VO_2_) and RER were measured in male and female control and UBC-SKO mice 4 weeks after tamoxifen treatment (n = 4 mice/group). (F) In male mice, serum T4 and T3 levels were measured in control and UBC-SKO mice (3rd cohort). For panels A-F, the data were analyzed by unpaired t-test. All data were shown as the means±SEM, and the p-values were shown as; ****, p< 0.0001; ***, p< 0.001; **, p< 0.01; *, p< 0.05. The 3^rd^ male cohort had n = 6–8 mice/group, and the female cohort had n = 8–9 mice/group.
(PDF)

## Abbreviations

*Acaca*: acetyl-CoA carboxylase alpha
Agrp: Agouti-related protein
*Aldh1a1*: aldehyde dehydrogenase family member A1
BAT: brown adipose tissue
*Bcl3*: B-cell lymphoma 3
*Cga*: chorionic gonadotropin alpha polypeptide
*Chrebp*: carbohydrate-responsive element-binding protein
*Cyp26a1*: cytochrome P450 family 26 subfamily A member 1
*Dhrs3*: dehydrogenase/reductase 3
*Dio 1*: iodothyronine deiodinase 1
ERT2 Cre: estrogen receptor T2 Cre recombinase
*Fasn*: fatty acid synthase
*Fitm1*: fat storage inducing transmembrane protein 1
*G6Pase*: glucose 6-phosphatase
*Glut4*: glucose transporter type 4
*Gmpr*: guanosine monophosphate reductase
*Gpd2*: glycerol-3-phosphate dehydrogenase 2
*Lrat*: lecithin retinol acyltransferase
Mc4r: melanocortin 4 receptor
*Myh6*: myosin heavy chain 6
*NCoR1*: nuclear receptor corepressor 1
*Pepck*: phosphoenolpyruvate carboxykinase
PGWAT: perigonadal white adipose tissue
*Pnpla2*: patatin like phospholipase domain containing 2
Pomc: proopiomelanocortin
*Rbp4*: retinol binding protein 4
*Rdh10*: retinol dehydrogenase 10
*Scd1*: stearoyl-CoA desaturase 1
SCWAT: subcutaneous white adipose tissue
*Serca1*: sarcoplasmic/endoplasmic reticulum Ca2+ transporting 1
*Smrt*: silencing mediator for retinoid and thyroid hormone receptors
*Srebp-1c*: sterol regulatory element-binding protein-1c
*Thrsp*: thyroid hormone responsive SPOT14
*Trhr*: thyrotropin releasing hormone receptor
UBC: ubiquitin C
*Ucp1*: uncoupling protein 1
α-MSH: α-melanocyte stimulating hormone
