# Supplementary figures and images for: Nuclear corepressor SMRT is a strong regulator of body weight independently of its ability to regulate thyroid hormone action
Source: PLoS One. 2019 Aug 12;14(8):e0220717. doi: 10.1371/journal.pone.0220717 (PMC6690520; doi:10.1371/journal.pone.0220717)

**A**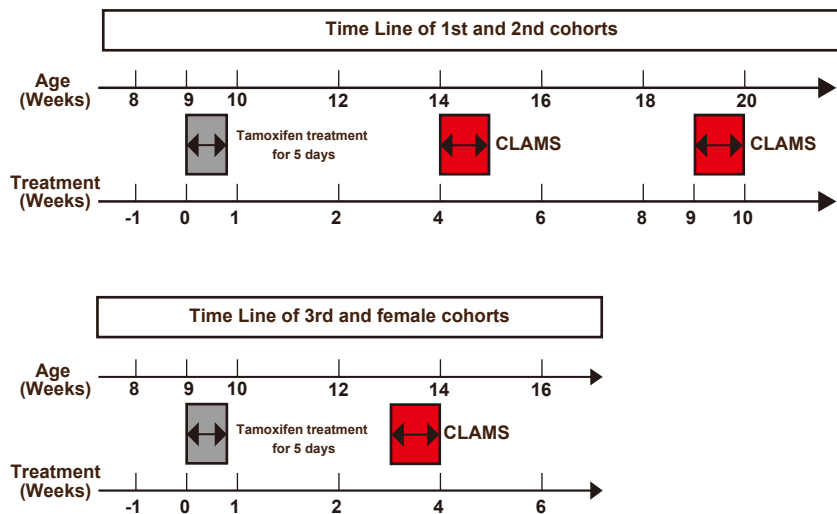**B**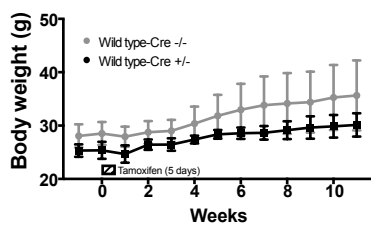**C**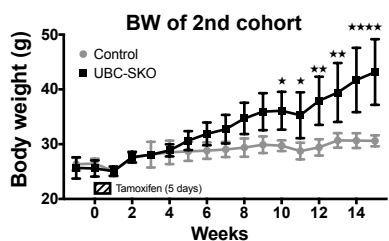**D**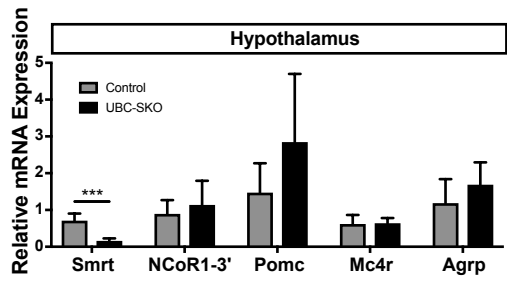**E**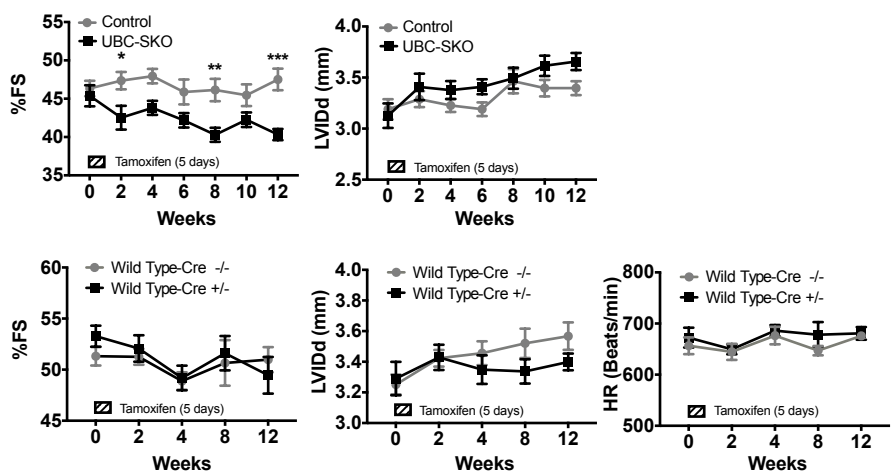

Supplement: S1 Fig — (A) A schematic representation of study design: tamoxifen treatments were performed in all mouse cohorts between 9–10 weeks of age. CLAMS studies were performed 3-4weeks (3rd male and female cohort), 4–5 weeks (1st and 2nd cohort), and 9–10 weeks (1st and 2nd cohort) after tamoxifen treatment. (B) Body weights were measured weekly in both wild type (WT) and WT-Cre male mice for 12 weeks following tamoxifen treatment (n = 6–8 mice/ group). (C) Body weights were measured weekly in control and UBC-SKO mice (2nd cohort). (D) Gene expression of Smrt, NCoR1-3’, Pomc, Mc4r, and Agrp in the hypothalamus were quantified by qPCR in control and UBC-SKO male mice at 12 weeks after tamoxifen treatment (2nd cohort mice). (E) Analyses of LVIDd, % fractional shortening (%FS), and heart rate (HR) using cardiac echocardiography were performed on control and UBC-SKO mice (upper panels, 1st cohort) and WT and WT-Cre (lower panels, n = 6–8 mice). For panels B, C and E, Two-way Repeated-Measures ANOVA was used, and for panel D, all qPCR data were analyzed by an unpaired t-test. Results are shown as the mean±SEM and p-values are; ****, p< 0.0001; ***, p< 0.001; **, p< 0.01; *, p< 0.05. 1st cohort included n = 6–7 mice/group, and 2nd cohort included n = 6–8 mice/group. (PDF) [file pone.0220717.s001.pdf]

## Pituitary

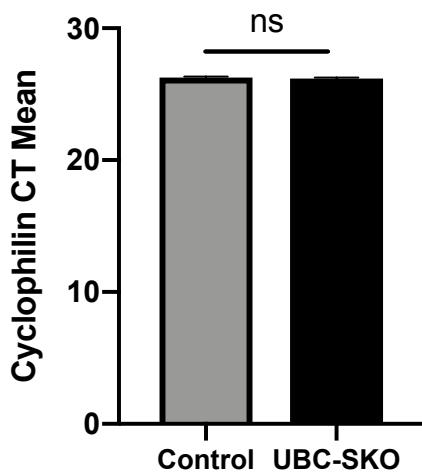

## PGWAT

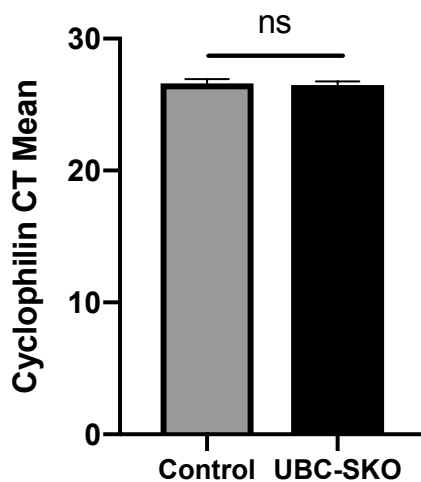

## Muscle

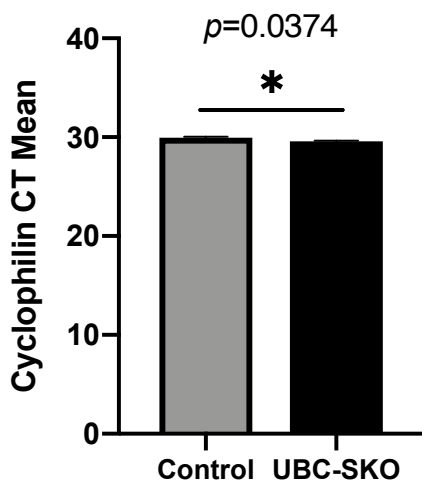

## Liver

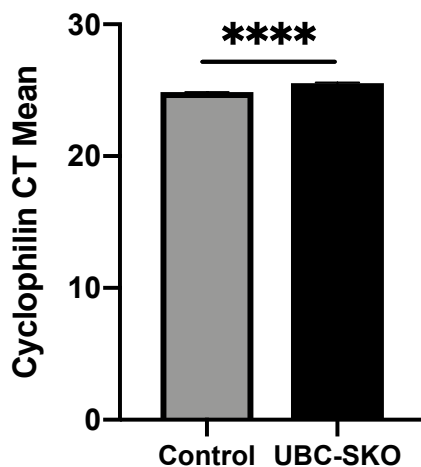

## Heart

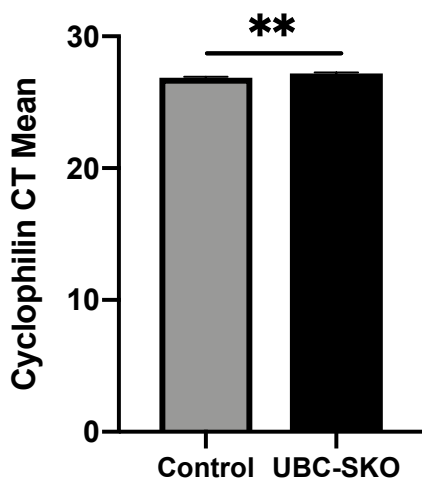

Supplement: S2 Fig — The mean of CT value was analyzed by qPCR in each tissue. Data were analyzed by unpaired t-test. Results are shown as the mean±SEM, and the p-values as; ****, p< 0.0001; **, p< 0.01; *, p< 0.05 (1st cohort, n = 6–7 mice/group). (PDF) [file pone.0220717.s002.pdf]

**A**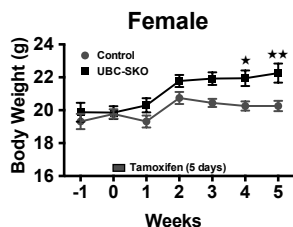**B**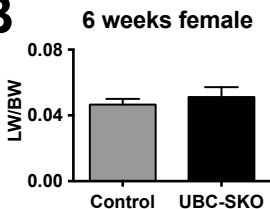**C**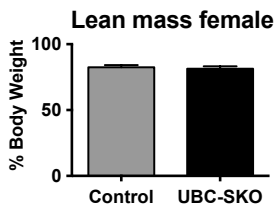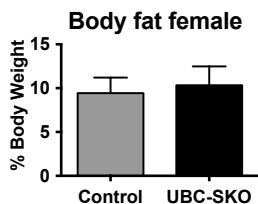**D**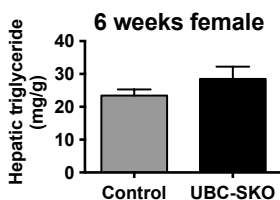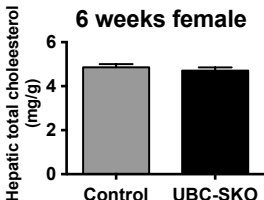**E**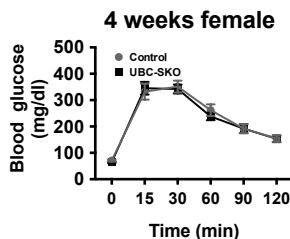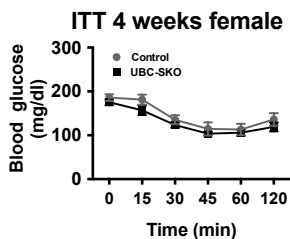**F**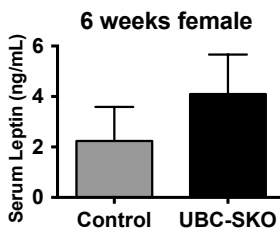

Supplement: S3 Fig — (A) Body weights were measured weekly in female control and UBC-SKO mice 6 weeks after tamoxifen treatment. (B) Liver weights, (C) body composition, and (D) hepatic triglycerides and cholesterol levels were measured in control and UBC-SKO mice. (E) Glucose tolerance and insulin tolerance tests were performed in female mice at 4 weeks after tamoxifen treatment. (F) Serum leptin levels were measured in control and UBC-SKO mice 6 weeks after tamoxifen treatment. All results are shown as the mean±SEM. For panels A and E, Two-way Repeated-Measures ANOVA was used, and the p-value is; **, p< 0.01; *, p< 0.05. For panels B, C, D, and F, the data were analyzed by unpaired t-test. The female cohort had n = 8–9 mice/group. (PDF) [file pone.0220717.s003.pdf]

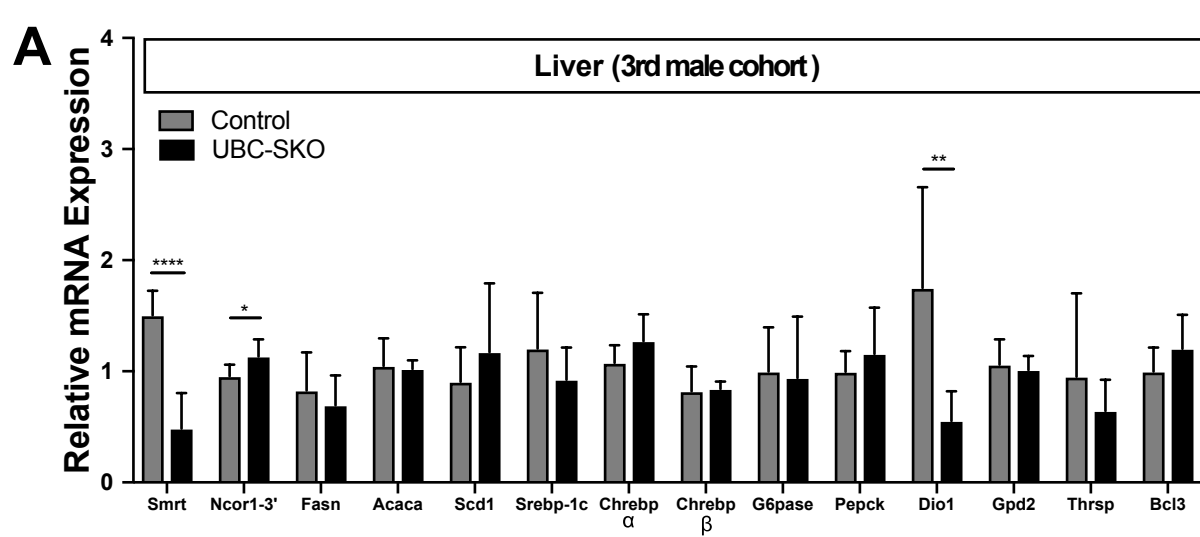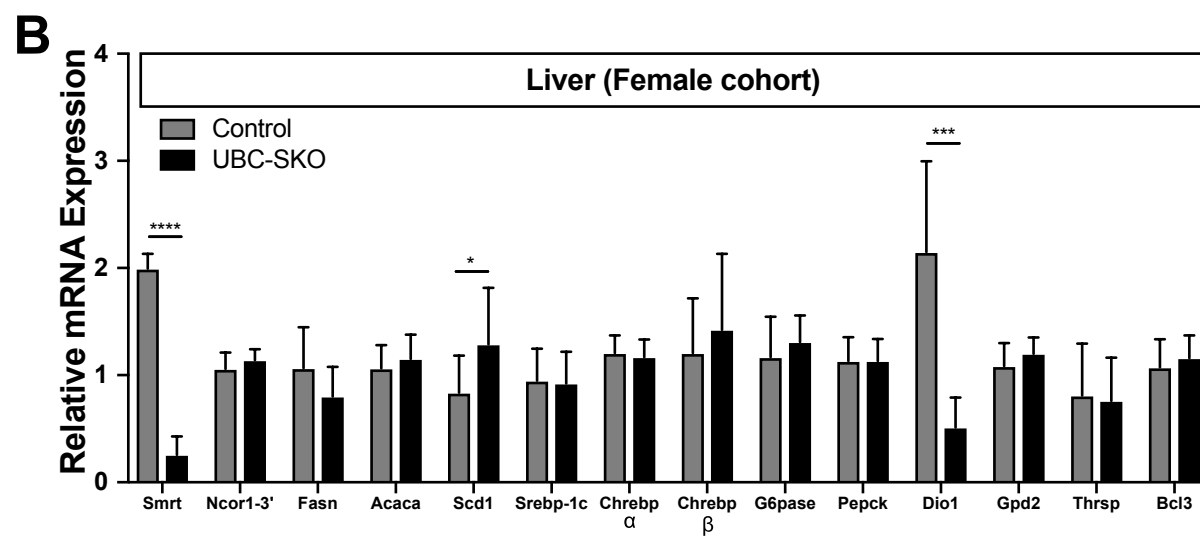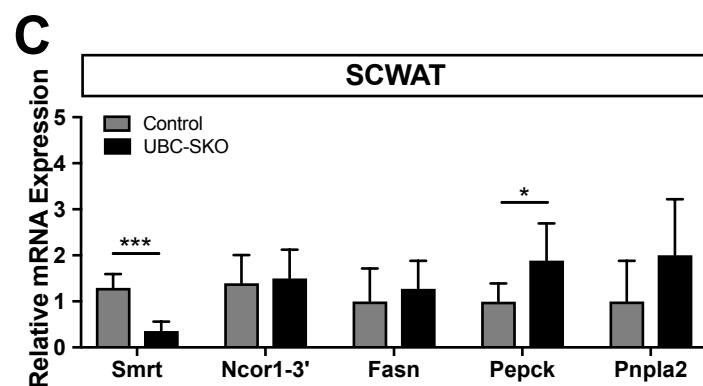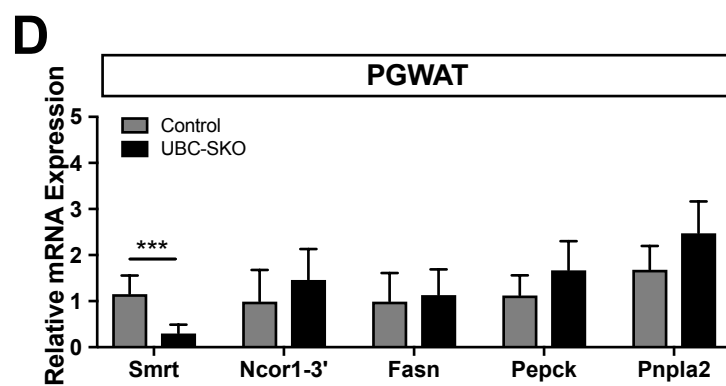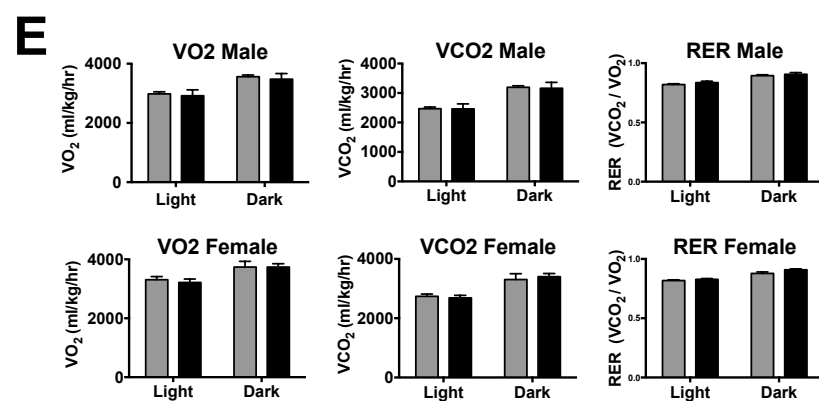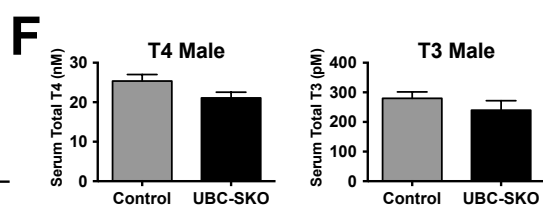

Supplement: S4 Fig — (A) In male mice, liver gene expression was measured in control and UBC-SKO mice 4 weeks after tamoxifen treatment (3rd cohort). (B) In female mice, liver gene expression in control and UBC-SKO female mice was assessed by qPCR 6 weeks after tamoxifen treatment (female cohort). (C) In male mice, gene expression in SCWAT was measured in control and UBC-SKO mice 4 weeks after tamoxifen treatment (3rd cohort). (D) In male mice, gene expression in PGWAT was measured in control and UBC-SKO mice 4 weeks after tamoxifen treatment (3rd cohort). (E) Carbon dioxide production (VCO2), oxygen consumption (VO2) and RER were measured in male and female control and UBC-SKO mice 4 weeks after tamoxifen treatment (n = 4 mice/group). (F) In male mice, serum T4 and T3 levels were measured in control and UBC-SKO mice (3rd cohort). For panels A-F, the data were analyzed by unpaired t-test. All data were shown as the means±SEM, and the p-values were shown as; ****, p< 0.0001; ***, p< 0.001; **, p< 0.01; *, p< 0.05. The 3rd male cohort had n = 6–8 mice/group, and the female cohort had n = 8–9 mice/group. (PDF) [file pone.0220717.s004.pdf]
